# Glial senescence enhances α-synuclein pathology owing to its insufficient clearance caused by autophagy dysfunction

**DOI:** 10.1038/s41420-024-01816-8

**Published:** 2024-01-26

**Authors:** Bin Hong, Yosuke Ohtake, Takahide Itokazu, Toshihide Yamashita

**Affiliations:** 1 Department of Molecular Neuroscience, Graduate School of Medicine, Osaka University, Suita, Japan; 2 Department of Neuro-Medical Science, Graduate School of Medicine, Osaka University, Suita, Japan; 3 WPI Immunology Frontier Research Center, Osaka University, Suita, Japan

**Keywords:** Parkinson's disease, Molecular neuroscience

## Abstract

Parkinson’s disease (PD) is characterized by the pathological accumulation of α-synuclein (α-syn) and loss of dopaminergic neurons in the substantia nigra. Aging is a significant risk factor for PD. The accumulation of senescent glial cells in the aged brain contributes to PD progression by inducing chronic neuroinflammatory processes. However, although the insufficient degradation of α-syn aggregates results in PD deterioration, the possible alteration in the ability of α-syn clearance in senescent glia has received little attention. In this study, we investigated how aging and glial senescence affect the capacity of α-syn clearance. We found that following the intra-striatal injection of human α-syn (hu-α-syn) preformed fibril, hu-α-syn pathology persisted more in aged mice compared with younger mice and that aged microglia exhibited greater accumulation of hu-α-syn than younger microglia. Moreover, in vitro assay revealed that the clearance of hu-α-syn was primarily dependent on the autophagy-lysosome system rather than on the ubiquitin-proteasome system and that the capacity of hu-α-syn clearance was diminished in senescent glia because of autophagy-lysosome system dysfunction. Overall, this study provides new insights into the role of senescent glia in PD pathogenesis.

## Introduction

Parkinson’s disease (PD) is an age-related neurodegenerative disorder characterized by the progressive loss of dopamine-producing neurons in the substantia nigra. It is associated with the pathological accumulation of misfolded α-synuclein (α-syn). Ninety-six percent of patients with PD are aged >50 years [1–3], and the disease affects 4% of the population aged >85 years [3]. Therefore, aging is considered the most critical risk factor for PD [4].

Cellular senescence is a biological state characterized by a persistent cell cycle arrest. It involves the expression of the cell cycle inhibitor p16INK4a and activation of damage-sensing signaling pathways, including p38/mitogen-activated protein kinase and nuclear factor-κB (NF-κB), considered molecular hallmarks. Cellular senescence increases the expression of senescence-associated secretory phenotypes (SASPs), such as proinflammatory cytokines, growth factors, and matrix metalloproteinases (MMPs) [5–8], actively promoting tissue alterations.

Accumulating evidence shows that cellular senescence plays a pivotal role in PD pathology. Chinta et al. reported that p16INK4a mRNA levels in the autopsied tissue of the substantia nigra pars compacta (SNpc) from patients with PD were higher compared with those in age-matched controls [9]. Riessland et al. revealed that dopaminergic neurons could become senescent with an increased SASP, which might lead to neuronal degeneration through local inflammation [10]. Additionally, neuronal senescence promotes SASP and subsequent α-syn secretion [11]. However, the contribution of glial senescence to PD pathology has also been reported. Astrocyte senescence during aging has been repeatedly demonstrated [12–14], and the number of senescent astrocytes increases in the SNpc of patients with PD [9]. Age-related microglial senescence has also been reported [15]. Although the causal relationship between glial senescence and PD pathology remains to be determined, it is widely accepted that neuroinflammation induced by glial cell-derived proinflammatory SASPs (e.g., interleukin [IL]-6, IL-8, and MMP3) is potentially involved in the exacerbation of neurodegeneration [5–8, 16]. Mitochondrial dysfunction is also reported in senescent glia [17], which promotes PD progression by producing oxygen-free radicals [17, 18]. Consistent with these findings, the depletion of senescent astrocytes/microglia by several senolytic drugs attenuates PD neuropathology in vivo [9, 19].

However, considering the involvement of glial cells in PD pathology, their role in the clearance of neuronal extrusions should be considered. Recent evidence demonstrates that cell-to-cell transmission of α-syn or α-syn aggregates is a key process in PD pathology [2, 20–22]. In particular, the experimental injection of fibrillar α-syn into the rodent brain leads to the spread of Lewy body-like pathology, a hallmark of PD [23–25]. Importantly, glial cells can ingest and degrade extracellular α-syn secreted from neurons [26–31], which can be beneficial for attenuating disease progression. Choi et al. demonstrated that microglia exerted a neuroprotective role by degrading neuron-derived α-syn via Toll-like receptor 4-NF-κB-p62-mediated selective autophagy [30], and Yang et al. showed that astrocytes significantly partially mitigated neuronal α-syn pathology by the phagocytic clearance of α-syn [31]. Therefore, reducing the glial cell-dependent α-syn clearance capacity may critically affect PD pathology. However, the effect of aging on this process has not been well-investigated.

In this study, we aimed to investigate how young and senescent glia respond to the α-syn challenge. We estimated the clearance of human α-syn (hu-α-syn) in young and aged mice following the intra-striatal injection of hu-α-syn preformed fibril (PFF) into the mouse brains. We established an in vitro senescent glial model to investigate the detailed mechanisms of hu-α-syn clearance in senescent glia. We treated young and senescent glia with hu-α-syn PFF. We provided evidence that (1) hu-α-syn pathology was largely deposited in aged mice 1 month after the intra-striatal injection of hu-α-syn PFF; (2) both young and senescent glia digested hu-α-syn, which was mainly dependent on the autophagy-lysosome system rather than on the ubiquitin-proteasome system (UPS); (3) hu-α-syn clearance was delayed in senescent glia because of impaired autophagic activity; and (4) aged microglia exhibited more extensive hu-α-syn pathology than younger microglia. Our findings reveal that hu-α-syn pathology tends to accumulate in the aged brain and that its clearance in senescent glia is delayed, resulting from the dysfunction of the autophagy-lysosome system.

## Results

### Aging promotes α-synuclein (α-syn) pathology after intra-striatal human α-syn preformed fibril (PFF) injection

**Fig. 1:**
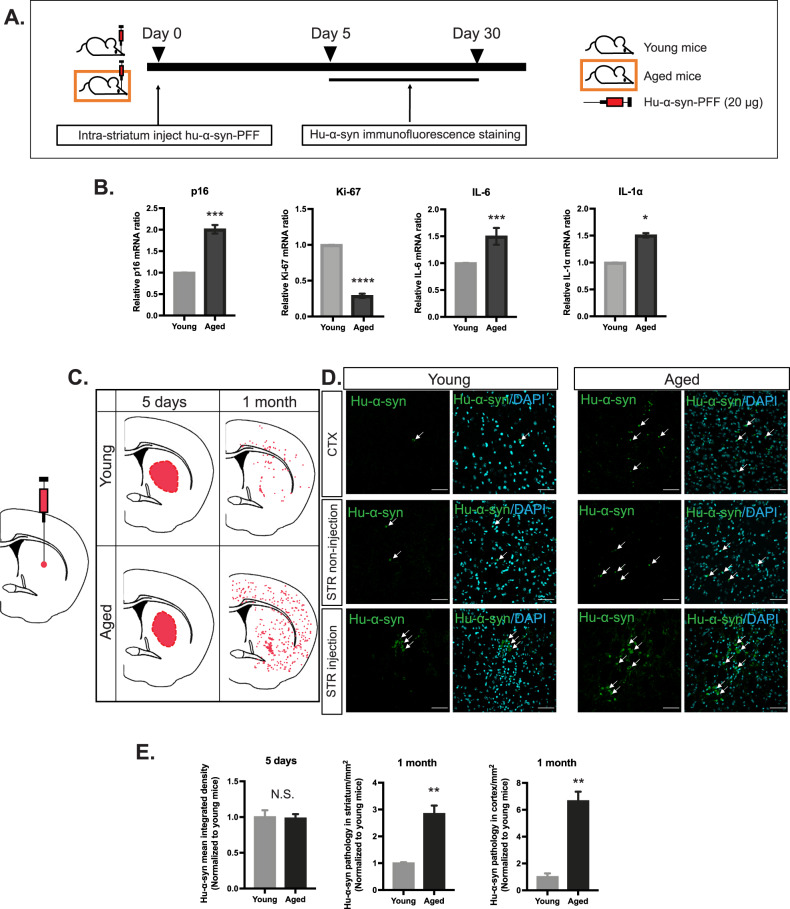
Summary of distribution of human-α-synuclein (hu-α-syn) pathology in young and aged mice following the intra-striatal injection of hu-α-syn preformed fibril (PFF). **A** The schema of young and aged mice intra-striatally injected with hu-α-syn PFF and analyzed after 5 days and 1 month, respectively, by detecting hu-α-syn pathology via immunofluorescence staining. **B** Real-time PCR results of cellular senescence markers (p16, Ki-67) and SASPs (IL-1α, IL-6) of young and aged mice (*n* = 6). **C** Following 5 days and 1 month post-injection, hu-α-syn diffused within the striatum (STR) of both young and aged mice. **D** Magnified representative image of hu-α-syn pathology distribution in injection site (STR injection, the remaining part of the STR [STR non-injection], cortex [CTX]) (scale bar: 50 μm). **E** Quantification and statistic results of hu-α-syn integrated density at 5 days and hu-α-syn pathology density in the STR and CTX at 1 month (n = 6). Values are presented as the means ± SEMs, and statistical significance was determined using the unpaired *t*-test *p < 0.05, **p < 0.01, ***p < 0.001, ****p < 0.0001. The arrow indicates hu-α-syn pathology.

First, we investigated how aging affects the clearance of α-syn pathology in the brain after the direct injection of hu-α-syn PFF into the striatum (STR) of young and aged mice (Fig. 1A). The mRNA levels of senescence markers (p16, Ki-67) and SASPs (IL-6, IL-1α) were confirmed in the aged brain before injection (Fig. 1B). Hu-α-syn PFF (10 μg/μl, 2 μl) or phosphate-buffered saline (PBS) was unilaterally injected into the STR of young and aged mice (medial-lateral [ML], +2.0 mm; anterior-posterior [AP], +0.2 mm; dorsal-ventral [DV], +3.0 mm). Subsequently, the distribution of hu-α-syn pathology at 5 days and 1 month post injection was assessed (Fig. 1A). At 5 days post injection, both in young and aged mice, hu-α-syn immunoreactivity was confined to the injection site and showed no difference between the groups (Fig. 1C, E left panel). However, after 1 month, hu-α-syn pathology widely spread through the brain in young and aged mice, with a significantly higher amount in aged mice than in young mice (Fig. 1C, E middle, and right panel). More specifically, hu-α-syn puncta were observed in the cortex (CTX) and STR (STR non-injection and STR injection) (Fig. 1D). These results demonstrate that hu-α-syn clearance is impaired in aged mice. We investigated whether α-syn clearance is impaired in senescent glia because microglia and astrocytes are the most critical cell types for misfolded protein clearance [26, 29, 30].

### Replicative senescence model of mixed glial culturing

**Fig. 2:**
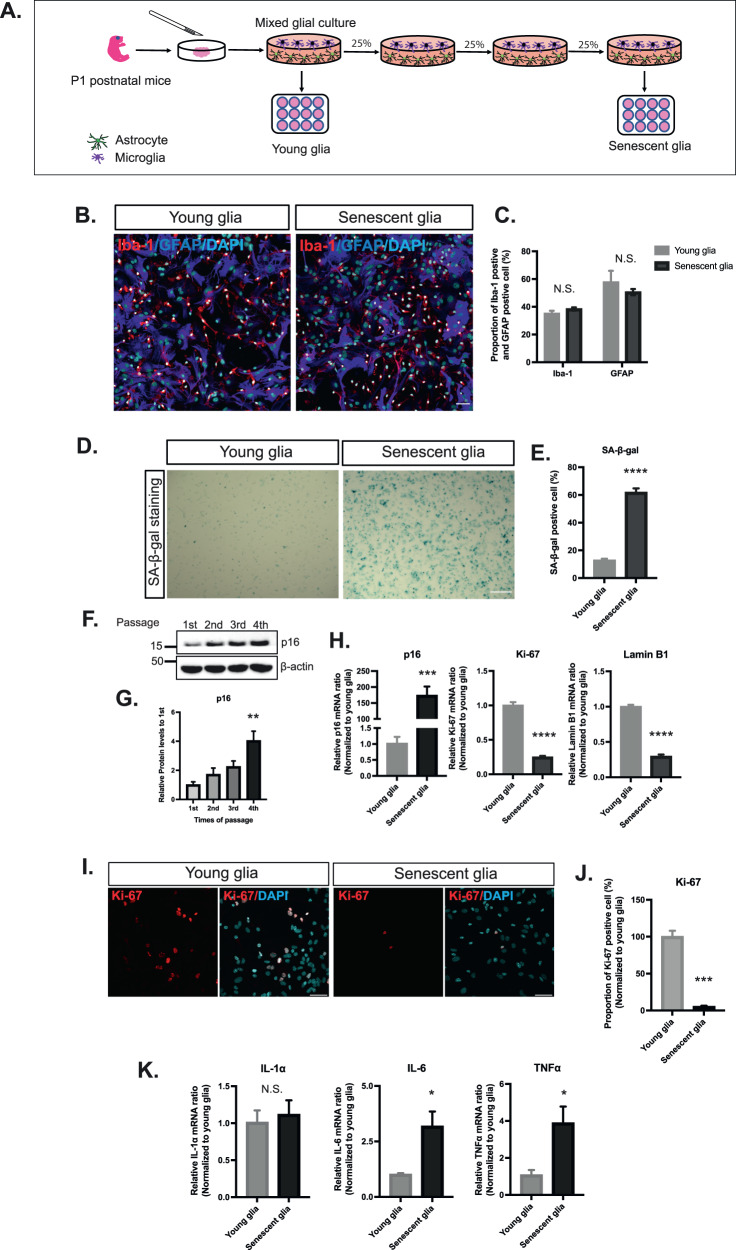
The establishment of replicative senescent glia. **A** Glia, obtained from P1 postnatal mice cortex, was seeded with a 1 × 10^5^ cells/cm^2^ density and grown until 100% confluence. For each passage, only 25% of the total cells were seeded. Passages were repeated thrice. Cells, considered to be young cells, were the cells before the first passage. In contrast, senescent cells were identified as those that finished three passages (completing the population doubling six times). **B** Representative images of the **C** proportions of the Iba-1-positive cell (microglia) and GFAP-positive cell (astrocyte) in young and senescent glia (*n* = 3) (scale bar: 50 μm). **D** SA-β-gal staining images of young and senescent glia (scale bar: 200 μm). **E** Quantification and statistical results of the SA-β-gal expression level (*n* = 8). **F** Representative western blotting image showing expression levels of p16 at each passage. **G** Quantification and statistical results of p16 expression level at each passage. **H** Real-time PCR result of three main cellular senescence markers (p16, Ki-67, lamin B1) for young and senescent glia (*n* = 6). **I** Representative Ki-67 immunofluorescence staining images (scale bar: 20 μm). **J** Statistical results of the proportion of Ki-67-positive cells in young and senescent glia (*n* = 6). **K** Real-time PCR results of SASPs (IL-1α, IL-6, TNF-α) (*n* = 6). Values are presented as means ± SEMs, and statistical significance was determined using the unpaired t-test (**C**, **H**, **J**, **K**), two-way ANOVA with Bonferroni test (**E**), and ordinary one-way ANOVA (**G**). *p < 0.05, **p < 0.01, ***p < 0.001, ****p < 0.0001.

we used an in vitro senescent glial culture model to explore the effects of aging on the clearance of hu-α-syn [32]. Briefly, the mixed glia was derived from the CTX of postnatal day 1 mice. It was passaged thrice, with 25% of the total harvested cells being used for each passage until 100% confluence was reached (Fig. 2A), resulting in quadrupling of the glia with each passage. After three passages, the glial cells underwent six rounds of division, resulting in a sixty-fourfold increase in cell number. The resulting mixed glia were considered senescent [33]. To provide context for the duration of the culture, our results indicated that approximately 45 days were required to obtain senescent glia. The young and senescent glia were mainly composed of astrocytes and microglia, as indicated by immunostaining against the microglia-specific marker ionized calcium-binding adapter molecule 1 (Iba-1) and astrocyte-specific marker glial fibrillary acidic protein (GFAP). Repetitive passages did not change their proportions (Fig. 2B, C). The three-step workflow of defining cellular senescence [33] was followed to verify the induction of cellular senescence: the senescent glia displayed an increased number of SA-β-gal-positive cells (Fig. 2D, E), elevated levels of p16 protein and mRNA (Fig. 2F, G, H left panel), decreased mRNA expression of lamin B1 (Fig. 2H right panel), decreased mRNA expression and proportion of Ki-67-positive cells (Fig. 2H middle panel, I, J), and increased mRNA expression levels of SASPs, including IL-6 and tumor necrosis factor (TNF)-α (Fig. 2K). These results indicate that replicative cell passaging can induce cellular senescence in primary glia, providing a suitable model for investigating hu-α-syn clearance in senescent glia.

### Human α-syn (hu-α-syn) clearance is delayed in senescent glia

**Fig. 3:**
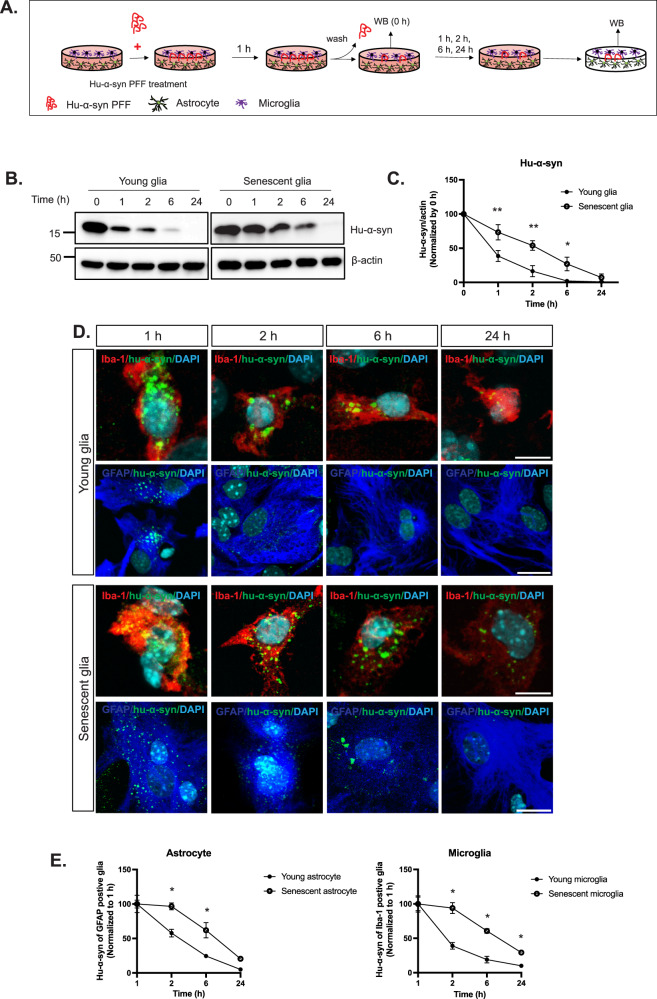
Hu-α-syn PFF clearance in young and senescent glia in vitro. **A** Glia was treated with hu-α-syn PFF for 1 h, washed out with PBS, replaced with fresh medium, and harvested at 0, 1, 2, 6, and 24 h after medium replacement. **B** Representative western blotting for remaining hu-α-syn and actin of young and senescent glia at 0, 1, 2, 6, 24 h following treatment with hu-α-syn PFF. **C** Quantification of remaining hu-α-syn of young and senescent glia at different time points (*n* = 4). **D** Representative images of hu-α-syn deposits in young and senescent glia at the different time points (scale bar: 10 μm). **E** Quantification and statistical results of hu-α-syn deposits in astrocytes and microglia of young and senescent glia (*n* = 6). Values are presented as means ± SEMs and statistical significance was determined by two-way ANOVA with the Bonferroni test. *p < 0.05, **p < 0.01.

Next, we investigated whether hu-α-syn clearance is impaired in senescent glia. We treated the mixed glia with hu-α-syn PFF (10 μg/ml) for 1 h, washed out hu-α-syn PFF in the medium, and cultured the cells with fresh medium for up to 24 h. We tested the amount of remaining hu-α-syn in the glial cells at 0, 1, 2, 6, and 24 h using western blotting (Fig. 3A) and found that at 1, 2, and 6 h, senescent glia contained more hu-α-syn than young glia (Fig. 3B, C). Specifically, although over 50% of the hu-α-syn engulfed by young glia was cleared within the first hour, only approximately 27% was digested in senescent glia during the same period. A similar phenomenon was observed at 2 h (84% in young glia versus 46% in senescent glia) and 6 h (98% in young glia versus 73% in senescent glia). However, by 24 h, >90% of hu-α-syn was cleared in both young and senescent glia (Fig. 3C). Immunocytochemistry confirmed that hu-α-syn deposits were observed in astrocytes and microglia and decreased over time (Fig. 3D). These results indicate that digestion by senescent astrocytes and microglia is slower than that by younger astrocytes and microglia (Fig. 3E). Overall, our findings demonstrate that both young and senescent glia can engulf and digest hu-α-syn PFF. However, the digestion process may be prolonged in senescent glia.

### Young and senescent glia clear hu-α-syn mainly via the autophagy-lysosome system rather than the ubiquitin-proteasome system (UPS)

**Fig. 4:**
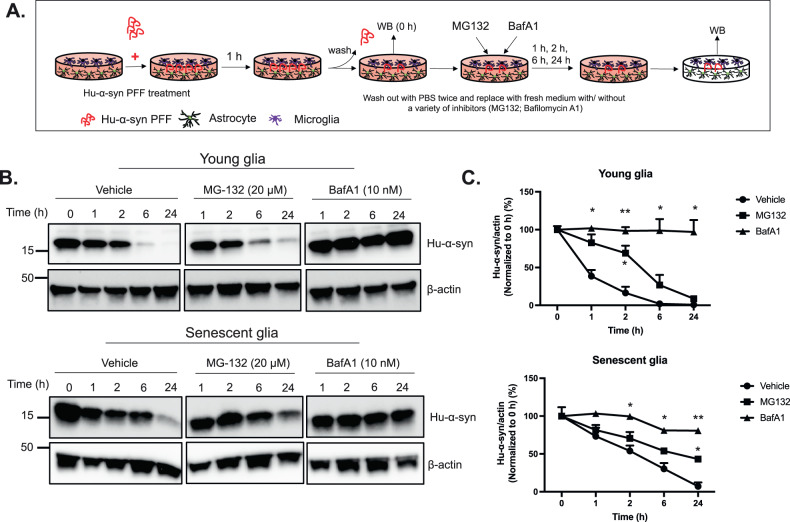
The autophagy-lysosome system plays a more important role in the degradation of hu-α-syn in both young and senescent glia than the ubiquitin-proteasome system. **A** The glia were treated with hu-α-syn PFF for 1 h, washed out with PBS, replaced with medium containing MG132 (20 μM) or bafilomycin A1 (BafA1) (10 μM), and harvested at 1, 2, 6, 24 h following medium replacement. **B** Representative western blotting of remaining hu-α-syn in young and senescent glia with MG132 and BafA1 at each time point. **C** Quantification and statistical results of remaining hu-α-syn in young and senescent glia under treatment of MG132 or BafA1 (*n* = 3). Values are presented as means ± SEMs and statistical significance was determined by two-way ANOVA with the Bonferroni test. *p < 0.05, **p < 0.01.

A previous study [34] reported that UPS and chaperone-mediated autophagy could target misfolded proteins in neurons in their monomeric or oligomeric form. In contrast, the aggregated form was directed to the lysosome via the autophagy-lysosome system. However, the dominant pathway of hu-α-syn clearance in glia is still not fully understood. We investigated this by employing two inhibitors, MG132 and bafilomycin A1 (BafA1), to inhibit the UPS and autophagy-lysosome system, respectively. Again, glial cells were exposed to hu-α-syn PFF for 1 h, washed out, and continuously cultured with the medium containing MG132 (20 μM) or BafA1 (10 μM) until 24 h (Fig. 4A). The remaining hu-α-syn was assessed using western blotting at 0, 1, 2, 6, and 24 h. The results showed that although MG132 could partially block hu-α-syn clearance at 2 h in young glia and at 24 h in senescent glia, BafA1 showed a stronger inhibitory effect on hu-α-syn clearance in both young and senescent glia. BafA1 delayed hu-α-syn clearance at all time points in young glia and at 2, 6, and 24 h in senescent glia (Fig. 4B, C). These data imply that although young and senescent glia digest hu-α-syn via the autophagy-lysosome system and UPS, the autophagy-lysosome system plays a more vital role than the UPS.

### Autophagic activity is impaired in senescent glia

**Fig. 5:**
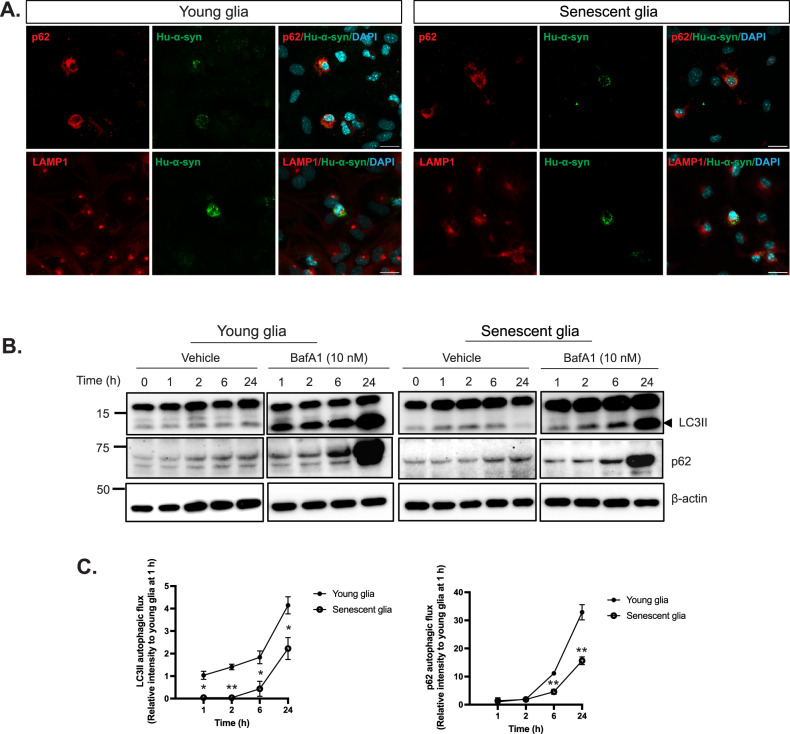
The autophagic flux of young and senescent glia after hu-α-syn PFF treatment. **A** Representative image of colocalization of hu-α-syn inclusion with p62 and LAMP1 in young and senescent glia 6 h after washing out (scale bar: 20 μm). **B** Representative western blotting of LC3B and p62 1 h after treatment of hu-α-syn PFF. The glia was incubated with or without BafA1 (10 nM) **C** Quantification and statistical results of LC3II and p62 autophagic flux (the difference of LC3II and p62 under BafA1 and no-BafA1 condition) (*n* = 3). Values are presented as means ± SEMs and statistical significance was determined by two-way ANOVA with the Bonferroni test. *p < 0.05, **p < 0.01.

We first examined whether hu-α-syn colocalized with p62 and lysosomal-associated membrane protein 1 (LAMP1) to investigate the mechanism underlying the delayed clearance of hu-α-syn in senescent glia. These proteins are involved in the autophagy-lysosome system (Fig. 5A). We also evaluated autophagic activity by measuring LC3II autophagic flux. Determining the number of autophagosomes alone (represented by LC3II) is insufficient to evaluate autophagic activity [35] because the accumulation of autophagosomes can indicate either autophagy induction or dysfunction in a later stage of the autophagy process, such as lysosome dysfunction or autophagosome-lysosome fusion blockage. Therefore, following the commonly accepted approach [35–37], we compared LC3II with and without BafA1, which blocks autophagosome-lysosome fusion. The difference in the intensity of the LC3II band between the vehicle- and BafA1-treated groups was used to estimate autophagic flux. We analyzed p62, a well-known autophagy-selective substrate, in the same manner (Fig. 5B). Our results showed that the difference in LC3II was significantly larger in young glia than in senescent glia at each time point and that the difference in p62 expression was considerably larger at 6 and 24 h (Fig. 5C). These findings demonstrate that autophagic activity is decreased in senescent glia, indicating that autophagic dysfunction in senescent glia may contribute to delayed clearance of hu-α-syn.

### Hu-α-syn pathology accumulates in aged microglia after 1 month of intra-striatal hu-a-syn PFF injection

**Fig. 6:**
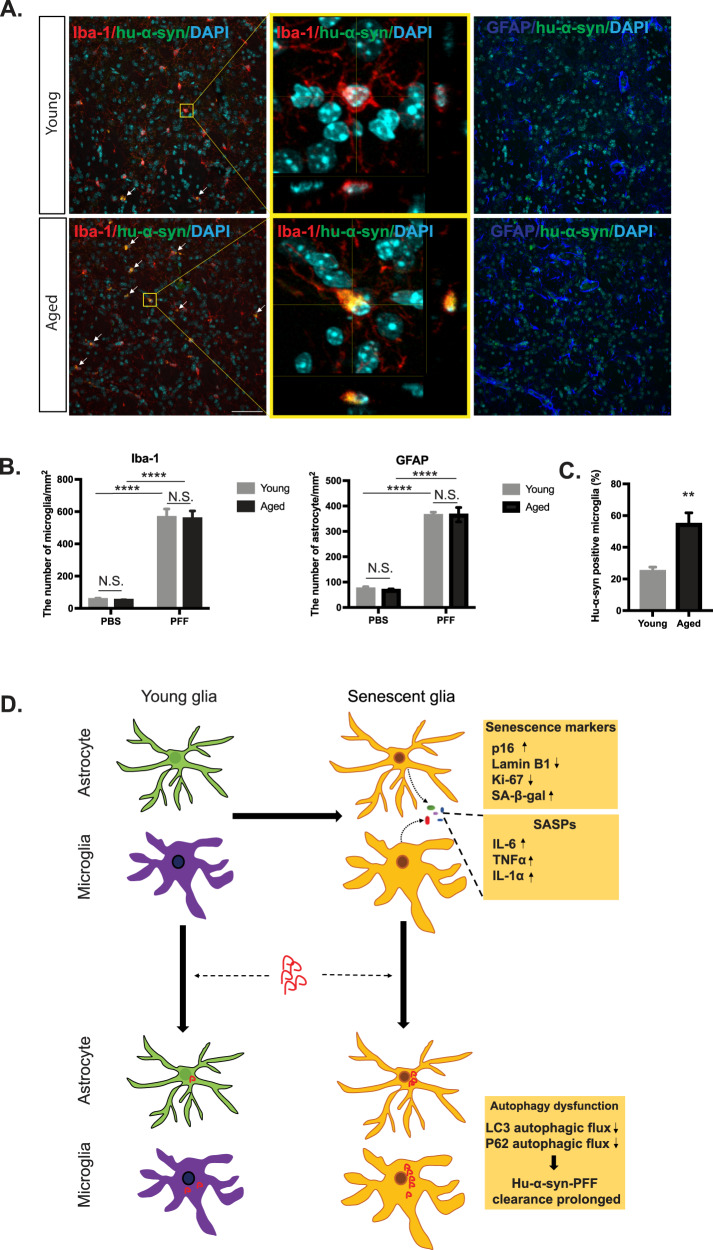
More hu-α-syn pathology accumulated in aged microglia after 1 month of intra-striatal hu-α-syn PFF injection. **A** Representative images showing the colocalization of hu-α-syn pathology and microglia (left) or astrocytes (right) in young and aged mice (scale bar: 50 μm). Brains were harvested 1 month after intra-striatal hu-α-syn PFF injection. **B** Quantification and statistical results of the percentage of hu-α-syn pathology-positive microglia in young and aged mice striatum (*n* = 6). **C** Quantification and statistical results of the number of microglia and astrocytes in young and aged mice after 1 month of intra-striatal hu-α-syn and PBS injection (*n* = 6). **D** Schematic picture illustrating that replicative glial passage induces replicative senescence, which shows characteristics of cellular senescence. Senescent glia shows prolonged hu-α-syn clearance via autophagy-lysosome system dysfunction. Values are presented as means ± SEMs and statistical significance was determined using the unpaired *t*-test (**B**) and two-way ANOVA with the Bonferroni test (**C**). **p < 0.01, ****p < 0.0001.

We evaluated the effects of hu-α-syn clearance in aged glia in vivo to obtain a deeper understanding of hu-α-syn deposition in aged brains. One month after hu-α-syn PFF injection, there was a significant increase in microglia and astrocytes in both young and aged mice, with no significant difference between the groups (Fig. 6A, B). Interestingly, hu-α-syn pathology was primarily observed in microglia but not in astrocytes (Fig. 6A). Strikingly, we found that aged mice had significantly more extensive hu-α-syn pathology in the microglia compared with young mice (Fig. 6C), indicating that hu-α-syn degradation may be impaired in aged microglia.

## Discussion

The molecular mechanism of the aging-related vulnerability of neurons has been intensively studied because aging is one of the most significant risk factors for PD [4]. However, the role of senescent glia in the clearance of α-syn has received little attention. As glia play a crucial role in clearing extracellular α-syn released from neurons [26, 28–31] and the accumulation of senescent glia in the aged mouse brain has been widely reported [38], we investigated whether and how α-syn clearance is affected by glial senescence. After confirming that the clearance of hu-α-syn pathology is delayed by aging in vivo, we addressed how senescence affects the ability of hu-α-syn digestion of glial cells. This exploration took advantage of an in vitro senescent glial culture model, revealing that senescent glia showed impaired hu-α-syn clearance capacity, likely because of autophagy-lysosome system dysfunction.

First, to investigate the effects of aging on the clearance of α-syn in vivo, we injected hu-α-syn PFF into the STR of young and aged mice. One month later, hu-α-syn puncta were detected in both young and aged mice, with a higher number detected in aged mice than in young mice. This observation agrees with previous findings by Nathalie et al. [2], who also reported that a higher density of α-syn pathology was detected in aged rats than in young rats following α-syn PFF injection into the gut wall [2]. In addition, we found that hu-α-syn deposition was frequently observed in the microglia in the aged brain, demonstrating that the efficient digestion of extracellular hu-α-syn PFF by glial cells, which may attenuate the aggregation of endogenous α-syn is impaired by aging.

We used an in vitro senescent glial culture model to investigate how senescence affects the ability of hu-α-syn digestion of glial cells. We compared the clearance efficiency of young and senescent glia using a one-pulse hu-α-syn PFF exposure strategy. Following temporal exposure to hu-α-syn PFF, the clearance of hu-α-syn was delayed by glial senescence (Fig. 3) within 24 h. Notably, after 24 h, only limited hu-α-syn was detected, even in senescent glia. This delay in clearance may be explained by the notion that, even if the clearance capacity is impaired in the senescent glial cells, it does not mean they have completely lost their digestion ability. In our experimental paradigm, glial cells are temporally exposed to pathological α-syn. Thus, senescent glia may have sufficient time to digest the engulfed hu-α-syn. This finding aligns with a previous study reporting that co-cultured microglia and astrocytes can promptly clear α-syn aggregates when receiving an α-syn PFF pulse. However, α-syn accumulation is prolonged when they are continuously treated with PFF [39]. These observations demonstrate that the balance between the ingestion and digestion capacities of glial cells is important for the clearance of α-syn aggregates. Thus, a senescence-related decrease in digestion capacity may critically affect the progression of α-synucleinopathy.

The delayed clearance of hu-α-syn in senescent glia raises questions about the dominant approach for digesting misfolded proteins in glia. The autophagy-lysosome system and UPS are critically involved in the pathology of neurodegenerative diseases [40] and complementarily and coordinately work with each other. However, their roles in senescent glia remain unclear. Our data revealed that both the UPS and autophagy-lysosome systems were involved in hu-α-syn degradation, and in young glia, UPS inhibition only partially impaired hu-α-syn degradation. Previous findings could explain this phenomenon that UPS inhibition leads to autophagy induction via the B-cell leukemia/lymphoma 2/beclin 1 (bcl2/BECN1) axis [41]. Thus, the autophagy-lysosome system can serve as a compensatory mechanism to mitigate UPS dysfunction by facilitating the degradation of ubiquitinated protein aggregates [42].

In contrast, we observed that inhibiting the autophagy-lysosome system in young glia almost entirely prevented hu-α-syn degradation. Considering the crosstalk between the UPS and autophagy, this result aligns with the concept that autophagy-lysosome system inhibition leads to the impairment of proteasomal flux, resulting in the dysfunction of proteasome activation [43]. Interestingly, in senescent glia, UPS inhibition by MG132 causes mild impairment of digestive capacity, in contrast to the effect of BafA1, which prevents hu-α-syn degradation at earlier time points (at 2, 6, and 24 h). Notably, the UPS is reduced in senescent cells [44]. Thus, it is possible that at baseline, the autophagy-lysosome system plays a dominant role in hu-α-syn clearance in senescent glia. This dominance of the autophagy-lysosome system in hu-α-syn clearance may explain our observation that UPS inhibition had a mild inhibitory effect on the degradation of α-syn aggregates. However, 24 h after the α-syn PFF challenge, while α-syn degradation was almost completed even with MG132 treatment in younger glia, α-syn deposition largely remained in senescent glia. This result demonstrates that compensation for UPS-dependent protein degradation by the autophagy-lysosome system is insufficient in senescent glia, possibly because of a decline in the autophagy-lysosome system capacity. Overall, these data show that the autophagy-lysosome system plays a crucial role in glial cell-dependent digestion of hu-α-syn, and decrease in the capacity of the autophagy-lysosome system in senescent glia results in insufficient compensation for UPS, thus leading to the exacerbation of α-syn pathology.

LC3 autophagic flux was used as a measure of autophagic activity to investigate further the autophagy-lysosome system in senescent glia (Fig. 4). Our results indicate that senescent glia show reduced autophagic activity at each time point, which aligns with recent evidence linking autophagy dysfunction and senescence. Yamamoto-Imoto et al. demonstrated that the transcription factor MondoA regulated cellular senescence by regulating the activity of autophagy [45]. Alvalo et al. [46] reported that disrupting the bcl2/BECN1 interaction enhanced autophagy, thereby preventing aging-related phenotypes. However, although the anti-senescence role of autophagy has been well-described, it also contributes to senescence establishment. Lee et al. revealed that selective autophagy regulated the stress support network of senescence by regulating protein stability [47]. Therefore, although the relationship between autophagy and senescence is complex and not fully understood, especially in glial cells, the impaired autophagy-lysosome system in senescent glia is evident, resulting in delayed clearance of hu-α-syn. Future studies are needed to unveil the detailed mechanism underlying the senescence-dependent decline in autophagy and how it affects the regulation of α-syn homeostasis within the central nervous system.

Finally, we examined the extent of clearance from aged glia in vivo. In the aged brain, microglia retained excessive hu-α-syn pathology compared with younger mice. This observation demonstrates lower efficiency of hu-α-syn clearance by microglia in aged brains, which is consistent with our in vitro data. In contrast, we found minimal hu-α-syn pathology colocalized with GFAP, possibly because α-syn is mainly engulfed not by astrocytes but by microglia. Additionally, because astrocytes transport hu-α-syn to nearby microglia through nano-tunnel secretion [48–50], 1 month is sufficient for astrocytes to digest and transport most of the internalized hu-α-syn to neighboring microglia.

In conclusion, our study found that hu-α-syn pathology in the aged brain was resistant to clearance and accumulation in aged microglia. Senescent glia show impaired hu-α-syn clearance, likely because of dysfunction of the autophagy-lysosome system. These findings demonstrate that senescent glia, which accumulates in aged brains, may contribute to the development of PD by secreting SASPs and by their reduced capacity to clear α-syn aggregates (Fig. 6D). Overall, our study provides new insights into the role of senescent glia in PD pathogenesis.

## Materials and methods

### Animals

Eight-week-old (young) C57BL/6J male mice and 85-week-old (aged) mice were purchased from CLEA Japan Inc. All experimental procedures, breeding, and housing were performed according to the Guidelines for Animal Care of Osaka University and were approved by the Care and Use of Laboratory Animals of Osaka University Graduate School of Medicine. No randomization or blinding was utilized in animal studies.

### Production of recombinant hu-α-syn PFFs

Hu-α-syn PFF was generated using monomeric hu-α-syn recombined by human full-length α-syn, as described previously. Briefly, *Escherichia coli* BL21 (DE3) (Invitrogen, MA, USA) was transformed with a pRK172 plasmid carrying the coding DNA sequence for hu-α-syn (kind gift from Dr. Virginia Lee, University of Pennsylvania, USA), incubated, and scaled up in Terrific Broth medium. *E. coli* pellets were collected by centrifugation at 4000 × *g* for 15 min and sonicated in a high-salt buffer containing protease inhibitors. The lysate was clarified by boiling for 20 min and centrifugation at 6000 × *g* for 20 min. The supernatant was dialyzed with 10 mM Tris overnight at 4 °C. Proteins were salted using 60% ammonium sulfate precipitation and spun down to the pellet. The pellet was dissolved in 10 ml anion exchange buffer, dialyzed overnight, purified using a HiTrap QFF column (GE Healthcare, Munich, Germany), and eluted with a 25 mM–1 M sodium chloride gradient. Eluted hu-α-syn was dialyzed using Slide-A-Lyzer Dialysis Cassette 10 K MWCO (Thermo Scientific, Grand Island, NY, USA) in dialysis buffer (150 mM potassium chloride [KCl], 50 mM Tris-hydrochloride [HCl], pH 7.5). The protein concentration was determined using a Pierce BCA Protein Assay Kit (Thermo Scientific, Grand Island, NY, USA). The hu-α-syn stock was dissolved in KCl-Tris (150 mM KCl, 50 mM Tris-HCl, pH 7.5) to a final concentration of 10 mg/ml for shock. Hu-α-syn PFF was generated by agitation at 1000 rpm in an orbital mixer (Eppendorf, Hamburg, Germany) at 37 °C for 10 days. Before each experiment, the hu-α-syn PFFs were sonicated using a sonicator for 5 s on ice and 5 s off for 2 min.

### Stereotaxic intra-striatal injection

Eight-week-old (young) and 85-week-old (aged) C57BL/6J mice were anesthetized with medetomidine (0.75 mg/kg), midazolam (4 mg/kg), and butorphanol (5 mg/kg) and secured in a stereotaxic frame (WPI, Sarasota, FL). In total, 2 μl PBS or hu-α-syn PFF (10 μg/μl) was intra-striatally injected at a speed of 0.67 μl per minute for each mouse with the following coordinates: ML, +2.0 mm; AP, +0.2 mm; and DV, +3.0 mm compared with bregma. The glass needle was maintained for 3 min after injection and gently removed from the brain. The mice were monitored for wound healing and recovery after the surgery.

### Replicative senescence model of mixed glial culturing

Primary mixed glia were obtained from the cortices of postnatal day 1 C57BL/6 J wild-type mouse pups (CLEA, Japan). Briefly, one pair of cortices was isolated on ice, dissociated with 0.25% trypsin at 37 °C 20 min, terminated by Dulbecco’s Modified Eagle Medium Nutrient Mixture F-12 with 10% fetal bovine serum, 100 mg/mL streptomycin, and 100 U/mL penicillin, and triturated into single-cell suspensions, which was filtered through a 100 μm cell strainer to clear cell debris or clumps. The mixed glia (1 × 10^5^ cells/cm^2^) were seeded to dishes pre-coated with 100 μg/ml poly-L-lysine (PLL) and grown until 100% confluence. The passages were repeated thrice. For each passage, only 25% of the harvested cells were seeded and grown until they reached 100% confluence. Cells considered young were observed after the first passage. In contrast, senescent cells were those below three passages (completing the population doubling six times). The cultures were maintained at 37 °C in a humidified 5% CO_2_. The culture media were changed by 50% in the first 24 h and thrice a week subsequently. Cells with a density of 5 × 10^4^/well were seeded on a PLL-pre-coated 12-well plate 24 h before hu-α-syn PFF treatment (10 μg/ml). After treatment with hu-α-syn PFF for 1 h, the cells were washed out with PBS and replaced with medium containing MG132 (20 μM) or BafA1 (10 μM). Dimethyl sulfoxide stocks were prepared from MG132 and BafA1 and stored at –80 °C after aliquot.

### Quantitative real-time polymerase chain reaction

Total RNA from the mouse CRX and mixed glia was obtained using an RNeasy Mini kit (QIAGEN). cDNA was synthesized via RNA reverse transcription using a cDNA synthesis kit (Thermo-Fisher). SYBR Green PCR Master Mix was used for target gene detection. The cDNA products were amplified using QuantStudio 7 Flex (Applied Biosystems). The following real-time polymerase chain reaction (PCR) conditions were used: 95 °C for 2 min, 40 cycles of 95 °C for 15 s, 60 °C for 20 s, and 95 °C for 15 s using the following primer pairs:

Glyceraldehyde-3-phosphate dehydrogenase (GAPDH) forward: 5’-GATCAACACGTACCAGTGCAA-3’

GAPDH reverse: 5′-TGCCTCGATGGACAGATAGA-3′

TNF-α forward: 5′-GATCGGTCCCCAAAGGGATG-3′

TNF-α reverse: 5′-CCACTTGGTGGTTTGTGAGTG-3′

IL-6 forward: 5′-GACAAAGCCAGAGTCCTTCAGA-3′

IL-6 reverse: 5′-TGTGACTCCAGCTTATCTCTTGG-3′

Ki-67 forward: 5′-CTGGTCACCATCAAGCGGAG-3′

Ki-67 reverse: 5′-CAATACTCCTTCCAAACAGGCAG-3′

Lamin B1 forward: 5′-GCTGCTGCTCAATTATGCCA-3′

Lamin B1 reverse: 5’-CCGCCTCATACTCTCGAAGC-3′

IL-1α forward: 5′-AGGGAGTCAACTCATTGGCG-3′

IL-1α reverse: 5′-ACTGTAGTCTTCGTTTTCACTGT-3′

P16 forward: 5′-TGAATCTCCGCGAGGAAAGC-3′

P16 reverse: 5′-TGCCCATCATCATCACCTGAA-3′

Relative mRNA concentrations were estimated using the 2^−△△Ct^ method. All ΔCt values were normalized to those of GAPDH.

### SA-β-gal assay

SA-β-gal staining was performed using a senescence β-galactosidase staining kit (Cell Signaling #9860). Mixed glia were seeded in a 6-well plate and fixed with a fixative solution, and SA-β-gal activity was assessed for 24 h. The percentage of senescent glial cells was expressed as the ratio of blue cells observed under a light microscope to the total number of cells evaluated using 4’,6-diamidino-2-phenylindole (DAPI) co-staining.

### Immunofluorescence staining and immunocytochemistry

Striatal sections (30 μm) were coronally cut using a Leica Cryostat from 4% paraformaldehyde (PFA)-perfused, fixed, and dehydrated brains. The sections were blocked with 5% normal goat serum (NGS) and 0.1% Triton X-100 in Tris-buffered saline (TBS) for 1 h at room temperature (RT). They were incubated with primary antibodies in a blocking buffer at 4 °C overnight. The following primary antibodies were applied: hu-α-syn (LB509, Abcam, 1:500), Iba-1 (019-19741, WAKO, 1:500), and GFAP (Abcam 1:500). The slides were washed thrice in tris-buffered saline (TBS) with Tween 20, followed by incubation with fluorescent-conjugated secondary antibodies and DAPI at RT for 1 h.

The cells were fixed in ice-cold 4% PFA at RT for 15 min and washed twice with TBS. Permeabilization and blocking were performed with 10% NGS (Jackson Laboratory), 3% BSA, and 0.1% Triton X-100 in TBS for 1 h at RT. Subsequently, 5% NGS and 0.1% Triton X-100 in TBS were diluted and applied to cells overnight at 4 °C. The following primary antibodies were applied: hu-α-syn (LB509, Abcam, 1:500), Iba-1 (019-19741, WAKO, 1:500), GFAP (Abcam 1;500), LAMP1 (ab24170, Abcam, 1:2000), and p62 (5114 T, cell signaling, 1:500). Before incubation with secondary antibody, the cells were washed with TBS thrice. Secondary antibodies were applied to the cells at RT for 1 h. The cells were mounted after additional washing. Images of tissues and cells were obtained using a confocal microscope (FV3000; Olympus Life Science).

### Sodium dodecyl-sulfate polyacrylamide gel electrophoresis and western blotting

Cell lysates were obtained using a radioimmunoprecipitation buffer with a protease inhibitor cocktail (Roche), which was applied to the cell culture plate to lyse and scrape all cells. A BCA protein assay kit (Thermo-Fisher) determined the protein concentration. In total, 10 μg total protein was diluted with a loading buffer for each lane and loaded onto 12% gels in sodium dodecyl-sulfate polyacrylamide gel electrophoresis running buffer (Bio-Rad). Gels were trans-membered to polyvinylidene fluoride membranes (Bio-Rad) using Trans-Blot Turbo for 3 min. The membranes were blocked in Bullet Blocking One (Nacalai Tesque) diluted in TBS for 1 h at RT. Primary antibodies β-actin (#3700 s, cell signaling, 1:5000), hu-α-syn (MJFR1, Abcam, 1:10,000), microtubule-associated protein 1 light chain 3B (LC3B) (ab192890, Abcam, 1:2000), p62 (5114 T, cell signaling, 1:2000), and p16 (ab211542, Abcam, 1:2000) were incubated at 4 °C overnight. Horseradish peroxidase-coupled secondary antibodies were incubated for 60 min at RT. Finally, they were visualized using Pierce ECL plus western blotting substrate (Thermo-fisher). The Chemi Doc MP imaging system (Bio-Rad) conducted detection, and the exposure time was set to ensure the strongest band of interest occurred before overexposure.

### Image analysis and quantification

For calculating hu-α-syn pathology and hu-α-syn/Iba-1-positive cell in vivo, stained brain slices (30 μm) z-stack pictures (×40 magnification) were captured by confocal fluorescence microscopy (FV3000; Olympus Life Science) at multiple areas of the STR and CTX. For quantification of hu-α-syn pathology, three or four brain slices were used for each brain, and positive signals were counted using ImageJ after setting the threshold. More specifically, hu-α-syn signals from 5 days after the injection were calculated using integrated density, whereas hu-α-syn signals from 1 month after the injection were calculated with the analyzed particles. Hu-α-syn inside microglia was manually counted in a z-stack picture.

Confocal microscopy (FV3000; Olympus Life Science) was used to capture z-stack images at ×40 magnification to calculate hu-α-syn deposits in microglia and astrocytes in vitro. the ImageJ software was used to quantify hu-α-syn-, Iba-1-, and GFAP-positive areas to quantify the hu-α-syn clearance in microglia and astrocytes. Colocalized areas of Iba-1 and hu-α-syn or GFAP and hu-α-syn were quantified to limit the analysis to internalized hu-α-syn only.

### Statistical analyses

All data are reported as means±standard error of the means of at least three independent experiments. Student’s unpaired t-test was used to determine the statistical significance of the differences between the two groups. For multiple group comparisons, statistical analysis was performed by one-way analysis of variance (ANOVA) or two-way ANOVA with Bonferroni post-hoc test using GraphPad Prism 8.

### Supplementary information

Full images

## Data Availability

All the data relevant to this study are presented in the manuscript. The data supporting the findings of this report are available from the corresponding author upon reasonable request.
