# Supplementary material for: Glial senescence enhances α-synuclein pathology owing to its insufficient clearance caused by autophagy dysfunction
Source: Cell Death Discov. 2024 Jan 26;10:50. doi: 10.1038/s41420-024-01816-8 (PMC10811334; doi:10.1038/s41420-024-01816-8)

Original image: Figure 2F

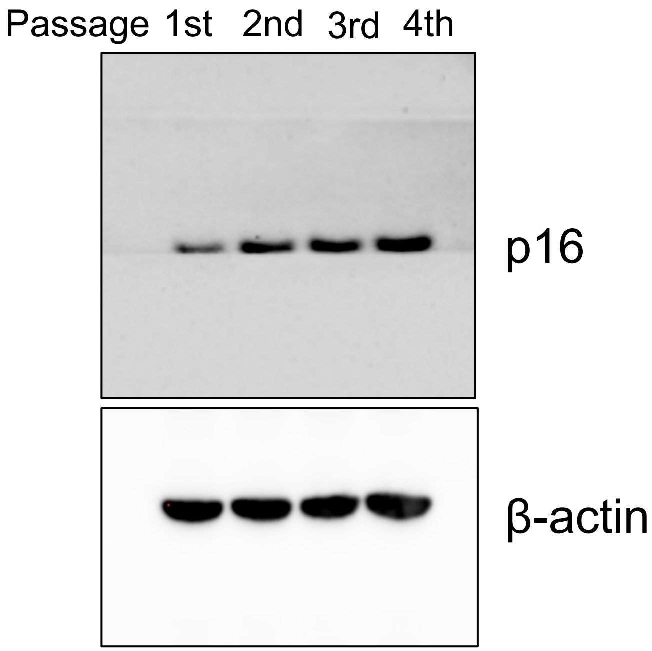

Original image: Figure 3B

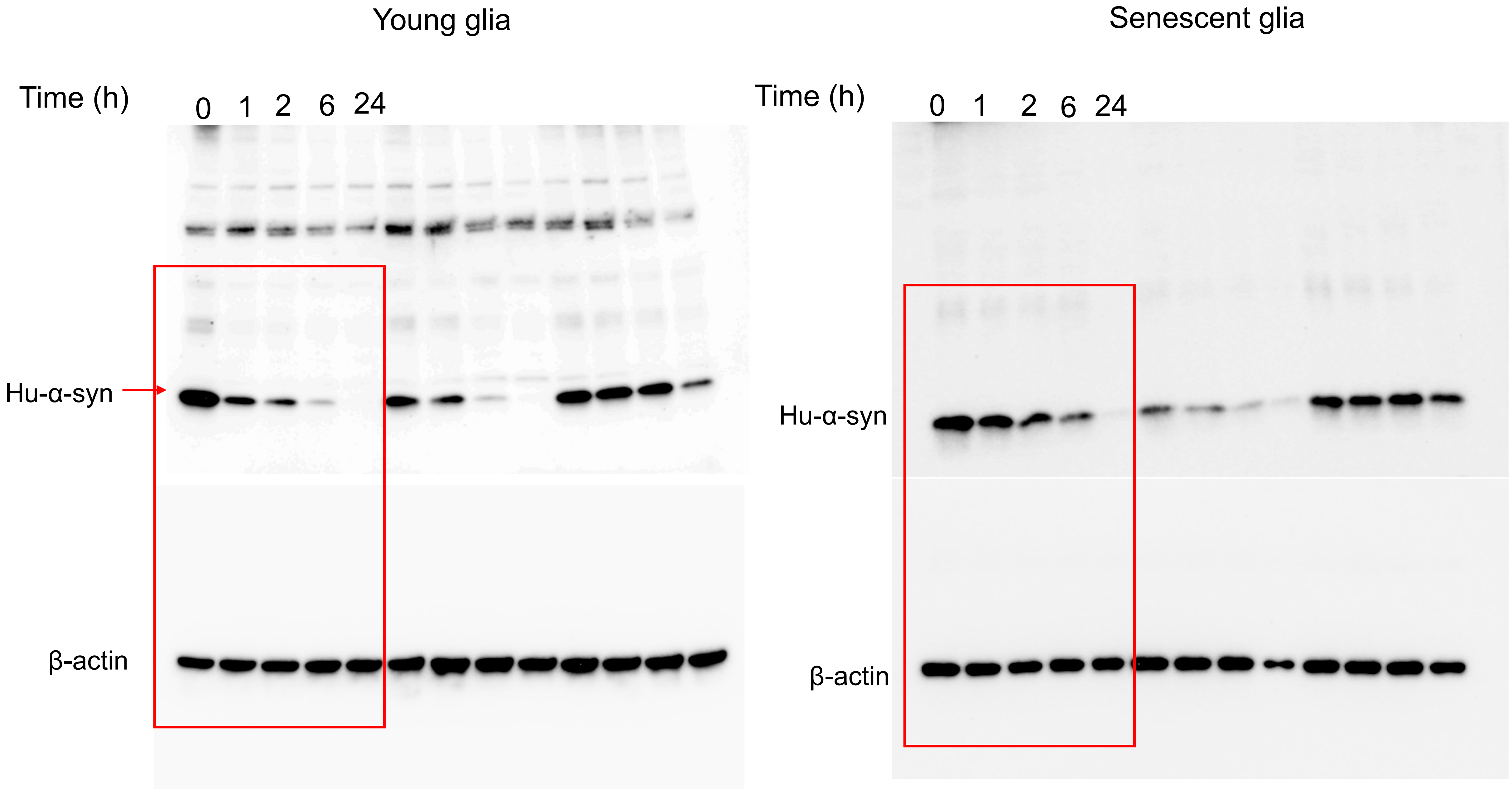

Original image: Figure 4B

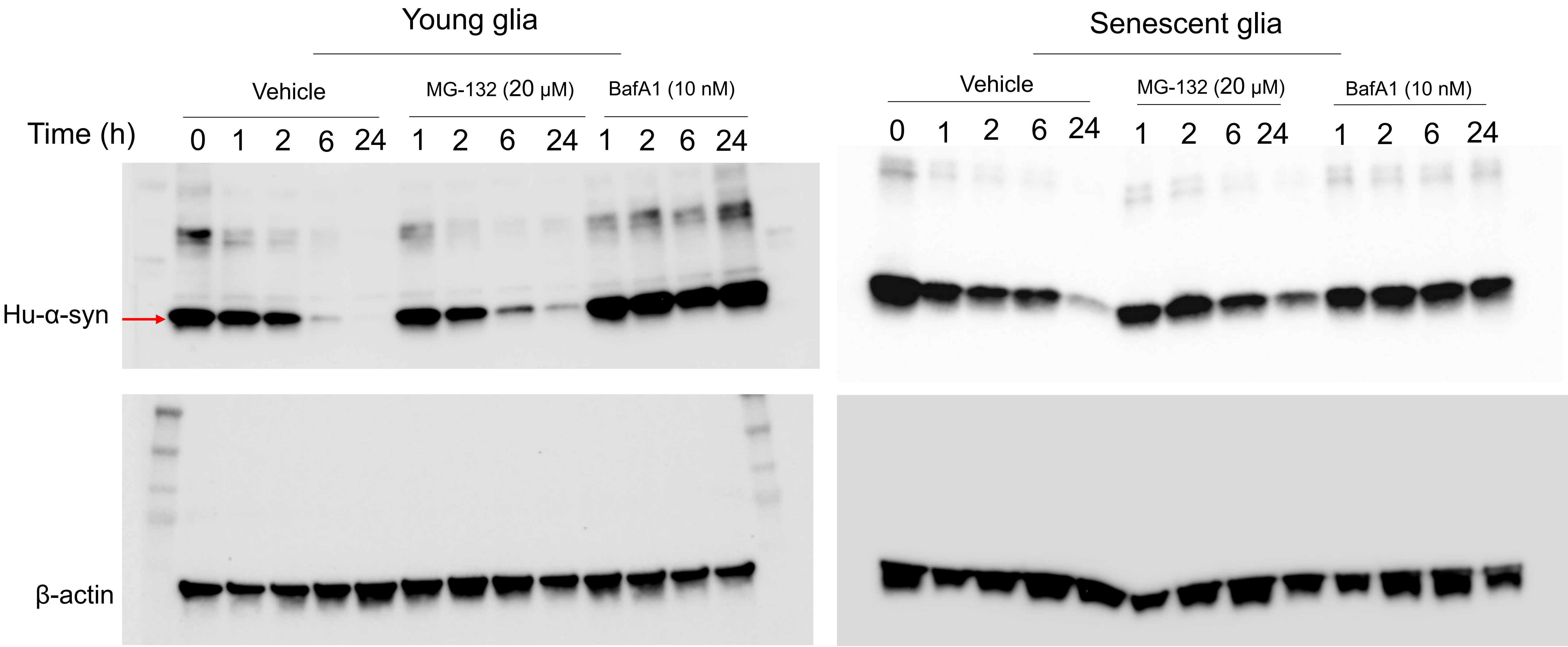

Original image: Figure 5B

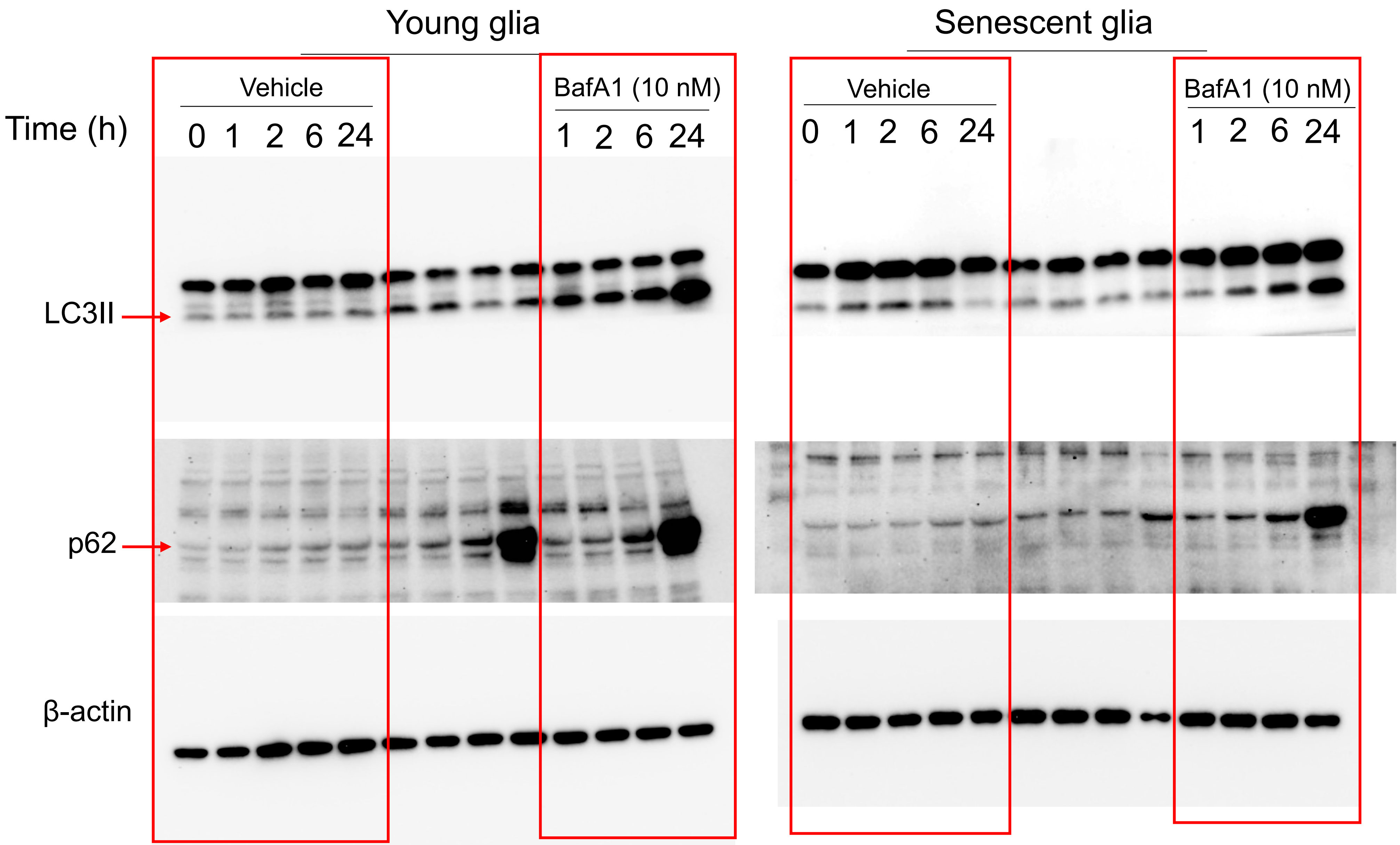

Supplement: Supplementary file 1 — Full images [file 41420_2024_1816_MOESM1_ESM.pdf]
